# Cryo‐Exfoliation Synthesis of Borophene and its Application in Wearable Electronics

**DOI:** 10.1002/advs.202502257

**Published:** 2025-04-04

**Authors:** Zhixuan Li, Gaurav Pandey, Arkamita Bandyopadhyay, Kamlendra Awasthi, John V. Kennedy, Prashant Kumar, Ajayan Vinu

**Affiliations:** ^1^ Global Innovative Centre for Advanced Nanomaterials School of Engineering College of Engineering Science and Environment The University of Newcastle Callaghan NSW 2308 Australia; ^2^ Malaviya National Institute of Technology Jaipur Jawahar Lal Nehru Marg, Jhalana Gram, Malviya Nagar Jaipur Rajasthan 302017 India; ^3^ Institut für Physik Theoretische Physik Martin‐Luther‐Universität Halle‐Wittenber 06120 Halle Germany; ^4^ National Isotope Centre 30 Gracefield Road, PO Box 30368 Lower Hutt Wellington 5040 New Zealand

**Keywords:** borophene, cryo‐exfoliation, nanogenerator

## Abstract

Borophene, an anisotropic Dirac Xene, exhibits diverse crystallographic phases, including metallic β₁₂, χ₃, and semiconducting α phases, alongside exceptional properties such as high electronic mobility, superior Young's modulus, thermal conductivity, superconductivity, and ferroelasticity. These attributes position borophene as a promising material for energy storage, electrocatalysis, and wearable electronics. However, its widespread application is hindered by existing synthesis methods that are expensive, complex, and yield‐limited. This study presents a novel, cost‐effective, environmentally friendly cryo‐exfoliation method for borophene synthesis. Crystalline boron powder is rapidly quenched in liquid nitrogen and subjected to mild sonication, producing borophene with lateral dimensions of ≈50 to 10 µm and few‐layer thicknesses. Advanced characterizations, including Atomic Force Microscopy (AFM), High‐Resolution Transmission Electron Microscopy (HRTEM), Raman Spectroscopy, and X‐ray Photoelectron Spectroscopy (XPS), confirm structural integrity, chemical purity, and minimal surface oxidation. Molecular dynamics simulations further elucidate the weakened inter‐layer coupling induced by cryo‐processing. The integration of borophene into Polyvinylidene Fluoride (PVDF) nanocomposites demonstrates its potential for wearable electronics, achieving motion‐sensitive devices with outstanding performance, generating output voltages up to ≈40 V. This scalable cryo‐exfoliation approach paves the way for borophene‐based applications in energy harvesting, sensing, and next‐generation electronics.

## Graphical abstract

This article introduces a novel cryo‐exfoliation method for synthesizing borophene, utilizing rapid quenching with liquid nitrogen and mild sonication to achieve scalable, high‐purity nanosheets. The study explores advanced characterizations, molecular dynamics simulations, and borophene‐based nanocomposites for wearable electronics, demonstrating its potential in energy harvesting and sensing applications through exceptional piezoelectric and triboelectric performance.

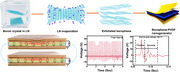

## 1 Introduction

Borophene, a member of the Xene family—mono elemental atom‐thin sheets—has emerged as a groundbreaking 2D material with an anisotropic crystal lattice. It exhibits exceptional properties, including high electronic mobility, superior Young's modulus, and remarkable thermal conductivity, often surpassing those of graphene along specific crystallographic directions^[^ ^1^, ^2^, ^3^, ^4^, ^5^ ^]^ These attributes arise from the quantum confinement of electrons in defect‐free single crystals, which endows borophene with unique physical and chemical characteristics, making it a superior candidate for next‐generation devices and sensors.^[^ ^6^, ^7^, ^8^ ^]^ Furthermore, borophene's extreme surface sensitivity allows for precise modulation of its electronic, photonic, thermal, and elastic properties, as well as its chemical reactivity, such as oxidation and corrosion.^[^ ^9^, ^10^, ^11^, ^12^, ^13^ ^]^

**Figure 1:**
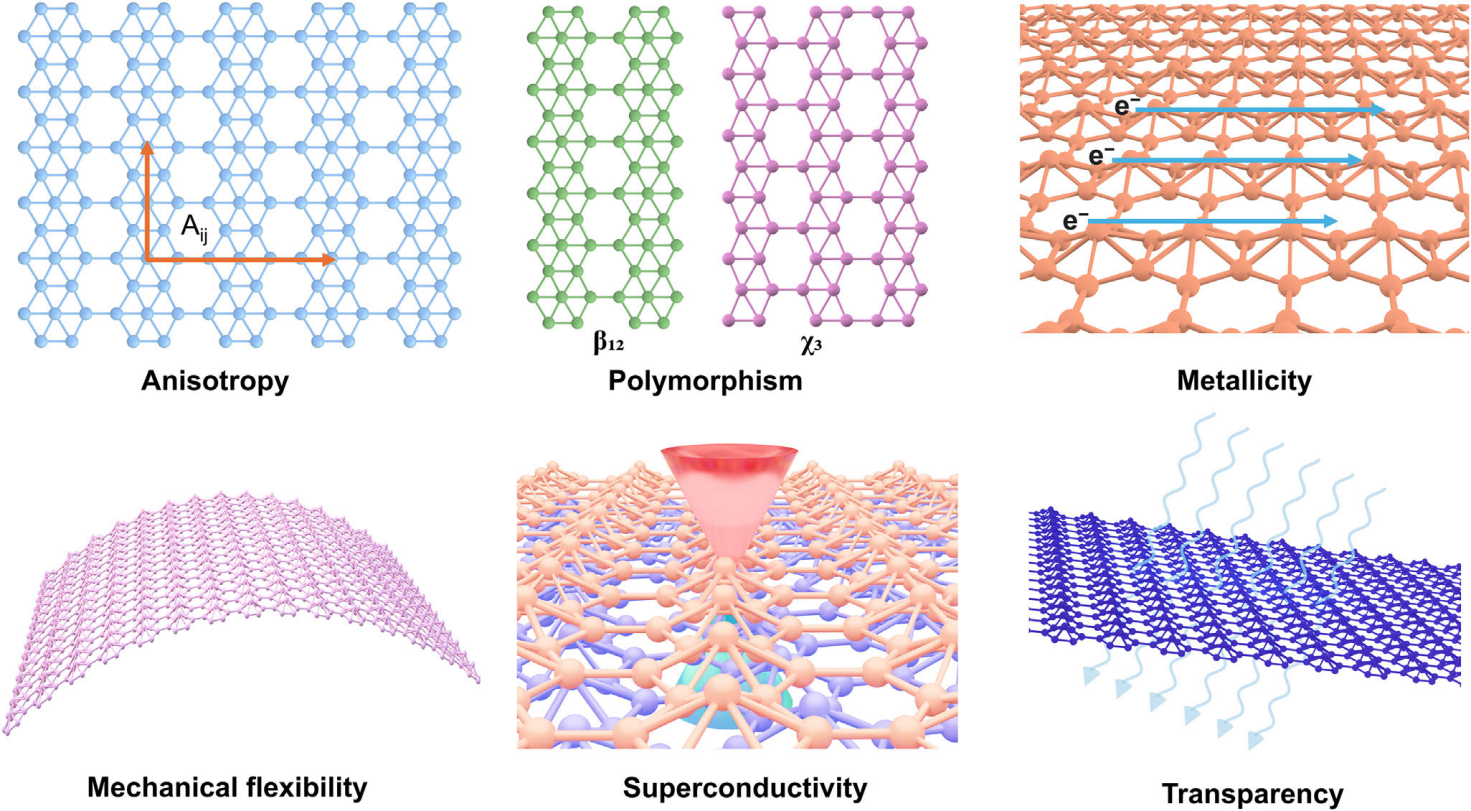
Schematic representations of borophene lattice structures and properties.^[^ ^21^ ^]^

Unlike other 2D materials, borophene exhibits a unique bonding configuration where two electrons are delocalized among multiple atomic centers (2e‐nc). This leads to the formation of diverse crystallographic phases, such as the metallic β₁₂ and χ₃ phases and the semiconducting α phase (>2 eV), each with distinct physical and chemical properties.^[^ ^14^, ^15^ ^]^ Thermal conditions during crystal growth determine how vacancies will align.^[^ ^14^ ^]^ These structural clones (phases) have distinct electron concentrations and distributions over the lattice, leading to variations in their electronic, optical, and mechanical behavior. The schematic representations in **Figure** **1** illustrate the diverse borophene lattice structures and their unique properties. Borophene exhibits distinguishable electronic, optical, and elastic behavior vis‐à‐vis other Xenes. Ridgelines in the β_12_ phase make it superior to others, especially due to its electronic mobility exceeding those of graphene along ridgelines. Moreover, physical properties show anisotropy, even in planar directions, due to anisotropic alignments of atoms. For example, while the elastic modulus is 401 nN/nm in the x direction, it is 337 nN/nm in the y direction.^[^ ^16^ ^]^ Additionally, borophene demonstrates a longer spin coherence length compared to other Xenes, enhancing its potential for applications in ferromagnetism and superconductivity.^[^ ^17^ ^]^ While graphene and phosphorene are not inherently electrochemically or catalytically active in their pristine forms, the metallic phases of borophene show significant promise in these areas. Recent advancements in borophene nanoarchitectonics, including substitutional doping,^[^ ^18^ ^]^ 2D–2D hybridization,^[^ ^19^ ^]^ and surface fluorination,^[^ ^20^ ^]^ have further expanded its electrochemical and catalytic capabilities. However, challenges remain in optimizing its performance through controlled defect generation, single‐atom doping, and hybridization with materials like MXenes and metallenes.

Borophene's potential extends across a wide range of applications, from ultrafast electronic chips and wearable devices to on‐the‐spot chemical/biomolecular sensing, energy storage, and catalysis. It is also a promising candidate for thermoelectric chips, electronic cooling, THz plasmonics, brain‐computer interfaces (BMI), and quantum computing. However, the realization of these applications hinges on the development of scalable synthesis methods that produce high‐purity borophene. Current synthesis techniques, such as atomic layer deposition (ALD), molecular beam epitaxy (MBE), and chemical vapor deposition (CVD), are limited by their high cost, complexity, and lack of scalability. Liquid‐phase methods, including sonochemical approaches and modified Hummer's method, often result in surface‐oxidized borophene, while micromechanical exfoliation suffers from layer inhomogeneity and high material costs. These limitations underscore the urgent need for innovative, scalable, and cost‐effective synthesis strategies.

Cryogenic exfoliation has recently emerged as a transformative approach for the scalable production of 2D materials. By leveraging the thermal stresses induced by liquid nitrogen treatment, this method significantly weakens interlayer interactions, facilitating the exfoliation of bulk materials into few‐layer nanosheets.^[^ ^22^, ^23^, ^24^, ^25^, ^26^, ^27^, ^28^, ^29^ ^]^ When combined with techniques like microwave‐assisted exfoliation, cryogenic treatment has demonstrated high‐yield production of 2D materials with superior purity.^[^ ^30^, ^31^ ^]^ Despite its potential, cryo‐mediated exfoliation of borophene has yet to be explored, presenting a timely opportunity to address the challenges of scalable borophene synthesis.

In this study, we report a novel cryo‐exfoliation method for synthesizing borophene. By rapidly quenching crystalline boron powder in liquid nitrogen, we induce thermal stress that weakens interlayer coupling, enabling the exfoliation of borophene sheets through mild sonication. The synthesized borophene is thoroughly characterized using advanced techniques, including AFM, HRTEM, Raman spectroscopy, and XPS. Molecular dynamics simulations provide further insights into the cryo‐exfoliation process, revealing the mechanisms behind the weakening of interlayer interactions.

The unique metallicity and flexibility of borophene make it an ideal candidate for flexible electronics, while its auxetic (negative Poisson's ratio) and ferroelastic properties open new avenues for exploration. To demonstrate its practical potential, we integrate cryo‐exfoliated borophene into PVDF nanocomposites for wearable electronics applications. These nanocomposites exhibit exceptional piezoelectric and triboelectric performance, generating output voltages of up to ≈40 V under mechanical deformation. This work not only establishes a scalable and environmentally friendly synthesis method for borophene but also highlights its potential in energy harvesting, sensing, and next‐generation electronics.

## 2 Results and Discussion

**Figure 2:**
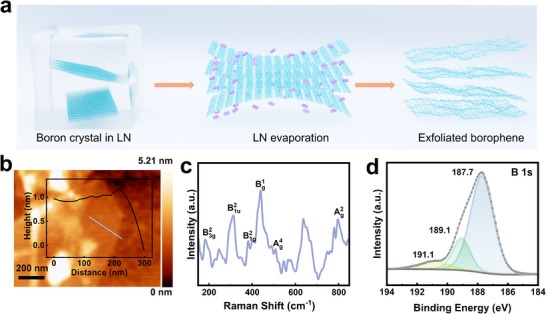
a) Schematic illustration of the synthesis process for cryo‐exfoliated borophene. b) An AFM image with a corresponding height profile is along the marked line. c) Raman spectrum of cryo‐exfoliated borophene, showing characteristic vibrational modes. d) XPS spectrum of the B 1s core level, highlighting chemical states and bonding environments in cryo‐exfoliated borophene.

Cryo‐quenching enables rapid physicochemical processing, facilitating a quick and environmentally friendly synthesis of borophene. Using liquid nitrogen (LN) and N‐methyl‐2‐pyrrolidone (NMP) during synthesis creates a reducing environment, ensuring the purity of the resulting material. Upon treatment with LN, the crystal volume of the boron contracts, rapidly trapping nitrogen molecules between the boron layers. This weakens the interlayer forces, making the atomic sheets of boron more susceptible to exfoliation into nanosheets (**Figure** **2**a). A solvent mixture of isopropanol (IPA) and NMP was employed during mild sonication. This combination increases the Hansen solubility parameters, enhancing the efficiency of sonochemical exfoliation. NMP prevents oxidation, while IPA facilitates easy drying of the material. AFM imaging revealed borophene sheets with lateral dimensions ranging from ≈50 to 3 µm and thicknesses from 0.4 to 3.2 nm, with a typical thickness of ≈1.2 nm (Figure 2b), indicating few‐layer sheets. Measurements from 10 samples over a 10 µm^2^ area showed an average roughness of 0.3 nm, consistent with monolayer borophene thicknesses of ≈0.27–0.31 nm reported by Mannix et al.^[^ ^1^ ^]^

Raman spectroscopy of the synthesized borophene sheets showed characteristic peaks at 182, 314, 383, 438, 508, and 798 cm⁻¹, corresponding to the B^2^_3_ _g_, B^2^_1u_, B^2^_1_ _g_, B^1^_g_, A^4^_g_, and A^2^_g_ vibrational modes,^[^ ^32^, ^33^, ^34^ ^]^ respectively (Figure 2c). These peaks confirm the formation of specific borophene phases, including the β₁₂ and χ₃ phases. Interestingly, the B‐O stretching peak at 191.1 eV in the B 1s XPS spectrum was significantly weaker than at 187.7 and 189.1 eV (Figure 2d). These B‐B peaks arise from distinct binding environments: tightly bound B atoms in ridgelines and loosely bound B atoms in the underlying layers.^[^ ^35^ ^]^ This high‐phase purity is rarely achieved with other liquid‐phase synthetic strategies, such as sonochemical methods,^[^ ^36^ ^]^ modified Hummer's methods,^[^ ^19^ ^]^ or electrochemical approaches.^[^ ^37^ ^]^

**Figure 3:**
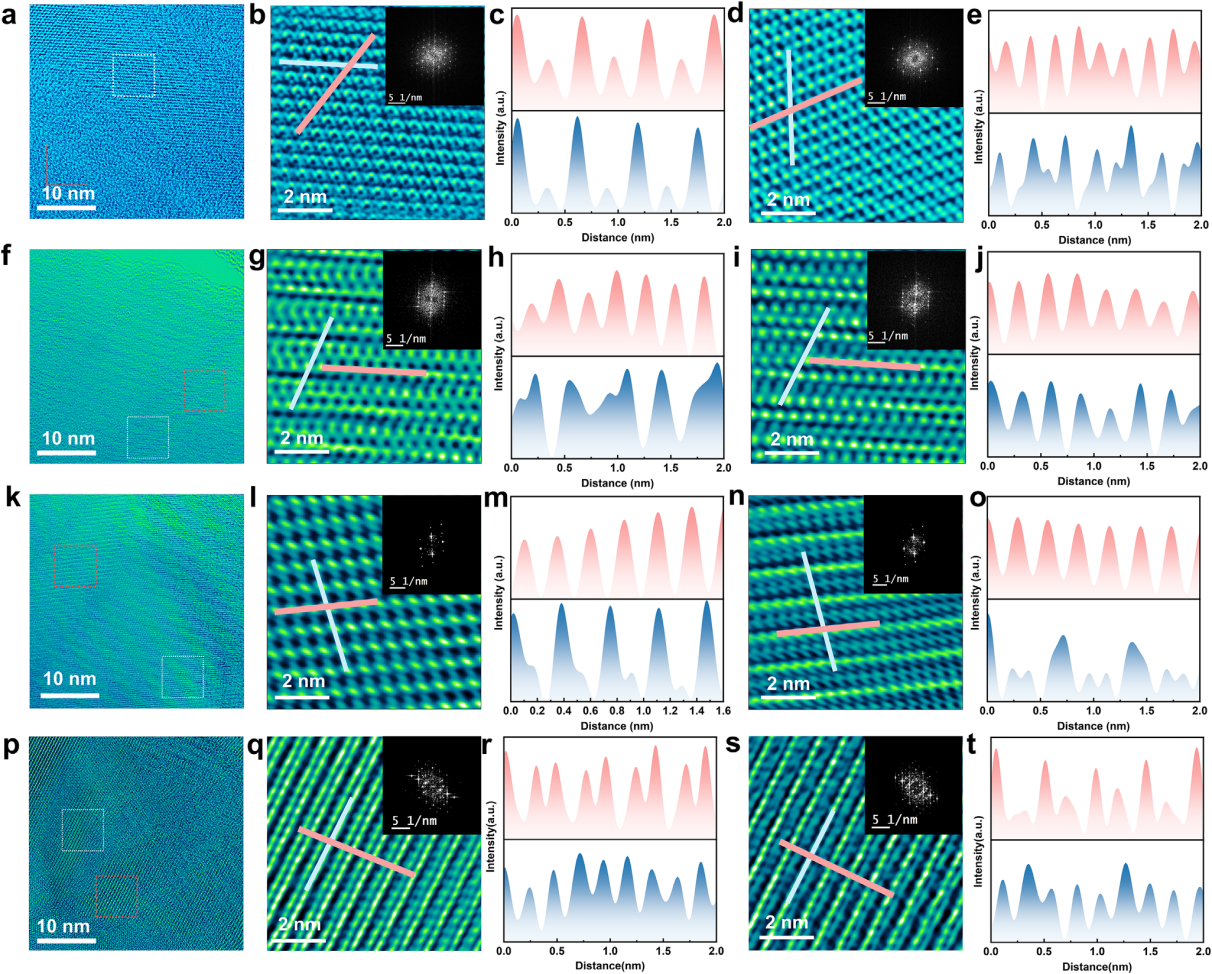
a,f,k) Cryo‐exfoliated borophene in three different areas. p) Sonochemical exfoliated borophene. b,g,l,q) Magnified images of the selected regions marked with white frames in (a, f, k, p), respectively. d,i,n,s) Magnified images of the selected regions marked with red frames in (a, f, k), respectively. Insets in (b,d,g,i,l,n,q,s) show corresponding Fast Fourier Transform (FFT) patterns. c,e,h,j,m,o,r,t) Line profiles of the red and blue lines indicated in (b,d,g,i,l,n,q,s) show periodic intensity variations along the marked directions.

HRTEM imaging revealed atomistic details of the crystallographic structures of cryo‐exfoliated borophene sheets, showcasing a variety of Moiré patterns, including translational, rotational, and combinatorial types. These patterns arise from stacking a few borophene layers with relative rotational misalignments and/or translational displacements. TEM images of three distinct regions of cryo‐exfoliated borophene samples are presented in **Figure** **3**a,f,k, with additional low‐magnification TEM images provided in (Figures S1–S7, Supporting Information). The images confirm that cryo‐exfoliation consistently produces large‐area, few‐layer borophene sheets while preserving their crystallographic integrity. Most of these sheets exhibit crystalline structures corresponding to the metallic β₁₂ or χ₃ phases, or a combination thereof. The relative rotation between stacked sheets creates arc‐like features, as evident in the magnified region (Figure 3b). Measurements of interatomic distances along two crystal symmetry directions revealed values of 0.33 and 0.29 nm (Figure 3c). In contrast, the adjacent region (Figure 3d) lacked circular arc features and exhibited interatomic distances of 0.24 and 0.31 nm (Figure 3e). Structural ripples, attributed to atomistic deformation, were observed in another region (Figure 3g), with average interatomic distances of 0.25 and 0.50 nm (Figure 3h). Intertwined structural configurations (Figure 3i) emerged due to local lattice strain, resulting in interatomic distances of 0.27 and 0.28 nm (Figure 3j) along symmetrical directions. These intertwined patterns suggest that borophene layers minimize strain by reorienting and reorganizing into lower‐energy configurations. Additionally, atomic twin clusters were identified (Figure 3l), with average interatomic distances of 0.30 and 0.36 nm (Figure 3m). Varieties of intertwined borophene crystal structures have been previously reported,^[^ ^38^ ^]^ with their formation tied to the conditions of substrate‐supported growth under high vacuum. The specific crystal structure that emerges depends on the compatibility between the lattice parameters and thermal expansion coefficients of borophene and the substrate. Mismatches in these properties generate interfacial strain, which promotes the formation of defects such as Stone‐Wales defects, vacancies, interstitials, and twinning. These defects are predominantly observed at grain boundaries.

Another prominent structural feature observed was atomistic ridgelines (Figure 3n), characteristic of the metallic β_12_ and χ_3_ phases of borophene. In these regions, the interatomic distances were measured to be 0.28 and 0.66 nm (Figure 3o). These findings highlight the structural diversity and dynamic reconfigurations of borophene, driven by local lattice strain and stacking configurations. For comparison, we included a sonochemically exfoliated borophene sample in Figure 3p. The zoomed‐in area in Figure 3q reveals two lines of atoms above and one line of atoms below, with the distance between the two upper lines measuring 0.18 nm. This arrangement forms a superlattice with a periodicity of 0.46 nm. The atomic distance along the upper lines is 0.25 nm (Figure 3r). Similarly, the zoomed‐in area in Figure 3s displays one line of atoms above and two lines of atoms below, resulting in a superlattice with a periodicity of 0.48 nm and an atomic distance along the upper line of 0.22 nm (Figure 3t). Due to the greater thickness of the sonochemical sample compared to the cryo‐exfoliated samples, Moiré patterns are not observed in these results.

Photographs documenting the liquid nitrogen evaporation process and the dispersion of cryo‐exfoliated borophene are provided in the Supporting Information (Figure S8, Supporting Information). Through batch processing, we can produce liters of borophene dispersion, making it suitable for various applications. Upon drying, we obtained 10–50 grams of borophene from multiple experimental batches. The production cost is significantly lower than that of conventional synthesis techniques, such as chemical vapor deposition or molecular beam epitaxy, because it avoids the use of chemical reagents and ultra‐high vacuum (UHV) systems. Additionally, this method is environmentally friendly, as it does not require toxic chemicals.

To further illustrate the advantages of cryo‐mediated exfoliation over other borophene synthesis methods, we provide a comparative analysis in **Table** **1**.

**Table 1:** Comparison of Cryo‐Mediated Exfoliation with Other Borophene Synthesis Methods.

Method	Exfoliation Medium	Temperature	Thickness	Lateral Size	Scalability	Yield	Crystallinity	Key Advantages	Limitations	Refs.
Cryo‐Mediated Exfoliation	Liquid nitrogen + solvent (isopropanol and NMP)	−196 °C to room temperature	0.4–3.2 nm	50 nm to 10 µm	High	Moderate	High	Scalable, cost‐effective, environmentally friendly, few‐layer boropene with minimal defects and high crystallinity	Requires cryogenic setup, solvent optimization	This Study
Mechanical Exfoliation	Adhesive tape	Ambient	Monolayer–few‐layer	<1 µm	Low	Very Low	Very High	Simple, no solvents needed, high‐quality flakes	Low yield, small lateral size, not scalable	[3]
Liquid‐Phase Exfoliation (LPE)	Organic solvents/water	Ambient–80 °C	3–10 nm	0.5–3 µm	Moderate	Moderate	Moderate	Scalable, solution‐processable, suitable for mass production	Defects from sonication, aggregation issues, solvent dependency	[19, 36]
Chemical Vapor Deposition (CVD)	Gaseous precursors (e.g., B₂H₆)	High (400–800 °C)	Monolayer–bilayer	10–100 µm	Low–Moderate	Low	High	Large‐area films, high crystallinity, control over layer number	High energy cost, substrate‐specific, slow growth rate	[5, 39, 40]
Molecular Beam Epitaxy (MBE)	Ultra‐high vacuum (UHV)	High (300–800 °C)	Monolayer	10–50 µm	Low	Very Low	Ultra‐High	Atomic‐level control, pristine interfaces, high‐quality single layers	Expensive, limited scalability, slow deposition rate	[1, 35]
Electrochemical Exfoliation	Electrolyte solution	Ambient–60 °C	2–8 nm	0.2–2 µm	Moderate	Moderate	Low–Moderate	Fast, low energy input, no need for high temperatures or vacuum	Risk of oxidation, defects, limited control over thickness	[37]
Modified Hummer's Method	Strong acids and oxidants	Controlled (room temperature to moderate heat)	Few‐layer to multi‐layer	Variable	Low	Low	Low	Can functionalize the material for specific applications	Harsh chemicals, low yield, high oxidation, not suitable for high‐purity boropene	[19]

**Figure 4:**
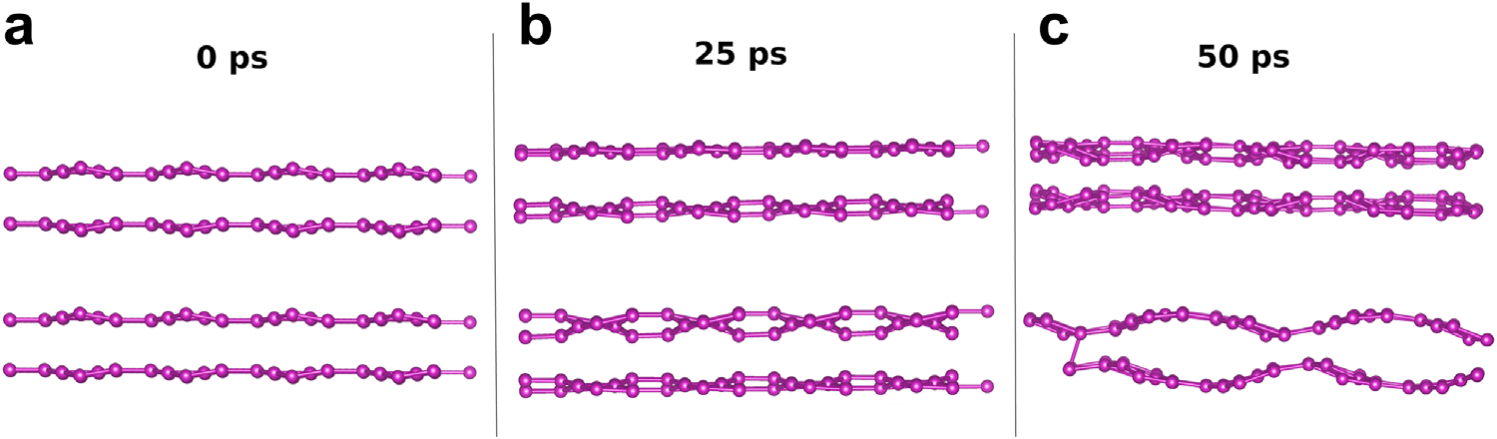
Molecular dynamics simulations showing the structural evolution of boron layers during cryo‐exfoliation over different time intervals: a) 0 ps, b) 25 ps, and c) 50 ps.

The system undergoes thermal shock during quenching, causing immediate stress and shrinkage. To alleviate this stress, the material forms various structural features. In cryo‐exfoliation, the system is rapidly cooled from room temperature to liquid nitrogen temperature in a fraction of a second, likely producing structural defects such as twins, corrugations, protrusions, ripples, and intertwined structures. Bulk boron crystals struggle to adapt to this abrupt thermal shock, exhibiting a non‐linear response. Cryo‐quenching induces atomistic structural changes, including crumpling and Z‐pinches (**Figure** **4**), and weakens inter‐layer coupling in boron layers, as evidenced by molecular dynamics simulations. Inter‐layer distances expand by 1.2 to 1.6 times their original values in the parent crystals before cryo‐quenching. Since inter‐layer coupling strongly depends on distance, a 1.6‐fold expansion reduces coupling by ≈17 times. This reduction stems from the material's stress relief mechanism under intense thermal stress from cryo‐quenching. The material responds to this stress on a rapid timescale. Molecular dynamics simulations reveal three distinct structural conformations at 0, 25, and 50 picoseconds (Figure 4a–c). The weakened inter‐layer coupling increases the layers' susceptibility to exfoliation. Following this, mild sonication for 30 min yields large‐area borophene sheets with high chemical phase purity. This work marks the first demonstration of cryo‐exfoliation for scalable borophene production, offering an effortless, reproducible, and cost‐effective method. Additionally, the approach is environmentally friendly, requiring no toxic chemical reagents.

**Figure 5:**
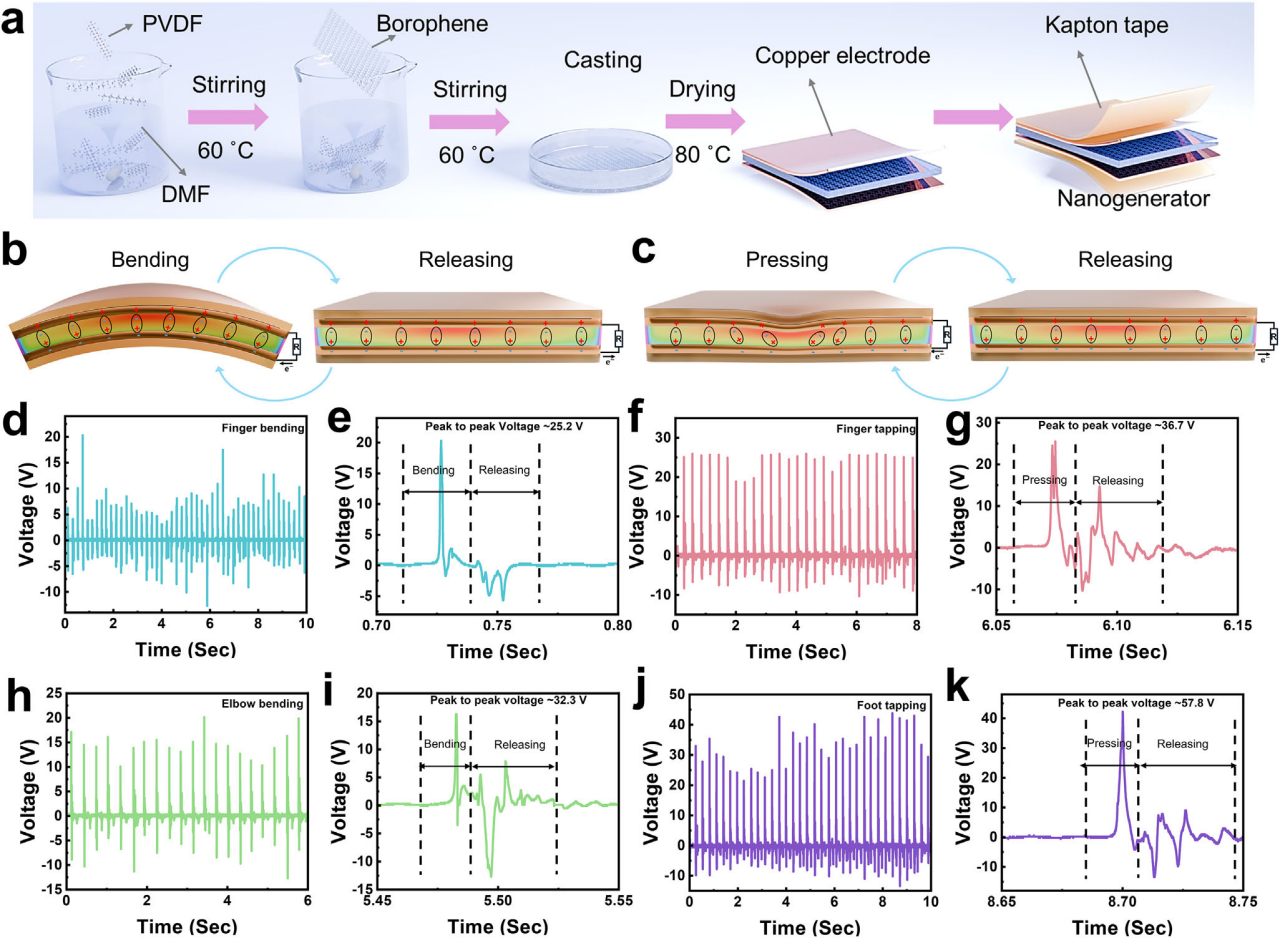
a) Schematic representation of the synthesis process for a borophene‐based piezo/triboelectric nanogenerator. b,c) Sequential illustrations depicting the bending, releasing, tapping, and releasing states of the nanogenerator during operation. d) Output voltage signals recorded during finger bending. e) The zoom‐in view of a single bending and releasing cycle for finger bending illustrates the peak‐to‐peak voltage. f) Voltage signals generated during finger tapping. g) Zoomed‐in view of a single tapping event, showing the peak‐to‐peak voltage for finger tapping. h) Voltage signals produced during elbow bending. i) Detailed view of elbow bending and releasing cycles. j) Voltage signals generated during foot tapping. k) Zoomed‐in view of a single pressing and releasing cycle, highlighting the peak‐to‐peak voltage for foot tapping.

The cryo‐exfoliated borophene‐based tribo/piezoelectric nanogenerator was fabricated by embedding borophene into a PVDF polymer matrix (**Figure** **5**a) through a low‐temperature synthesis and film‐casting process. The resulting nanogenerator exhibited excellent piezoelectric and triboelectric responses when subjected to mechanical deformations caused by body movements (Figure 5b,c). For instance, during finger bending, the device generated a peak‐to‐peak voltage of 25.2 V (Figure 5d,e). Similarly, finger tapping produced a peak‐to‐peak voltage of 36.7 V (Figure 5f,g). These results encouraged further investigations with elbow bending and foot tapping, yielding peak‐to‐peak voltages of 32.3 V (Figure 5h,i) and 57.8 V (Figure 5j,k), respectively. Integrating borophene within the PVDF matrix forms a conductive network that significantly enhances the electrical properties of the nanocomposite. When subjected to mechanical deformation, such as compression, tension, or bending, the polar PVDF molecules align along the applied stress, causing charge separation at the surface and generating an electric field. This process induces a detectable piezoelectric voltage at the electrodes attached to the nanogenerator. The triboelectric effect is activated when the composite interacts with a surface of differing electronegativity. This contact, often caused by friction or rubbing, facilitates charge transfer between the surfaces. The PVDF either gains or loses electrons, leading to surface charge accumulation. The embedded borophene further amplifies the triboelectric effect by providing a high surface area for contact and improving charge generation efficiency due to its excellent electrical conductivity. The synergy between the piezoelectric and triboelectric effects in the PVDF‐borophene composite results in efficient voltage generation. This energy can be harvested and utilized for energy storage or to power small electronic devices, demonstrating the potential of the nanogenerator for sustainable energy applications.

**Figure 6:**
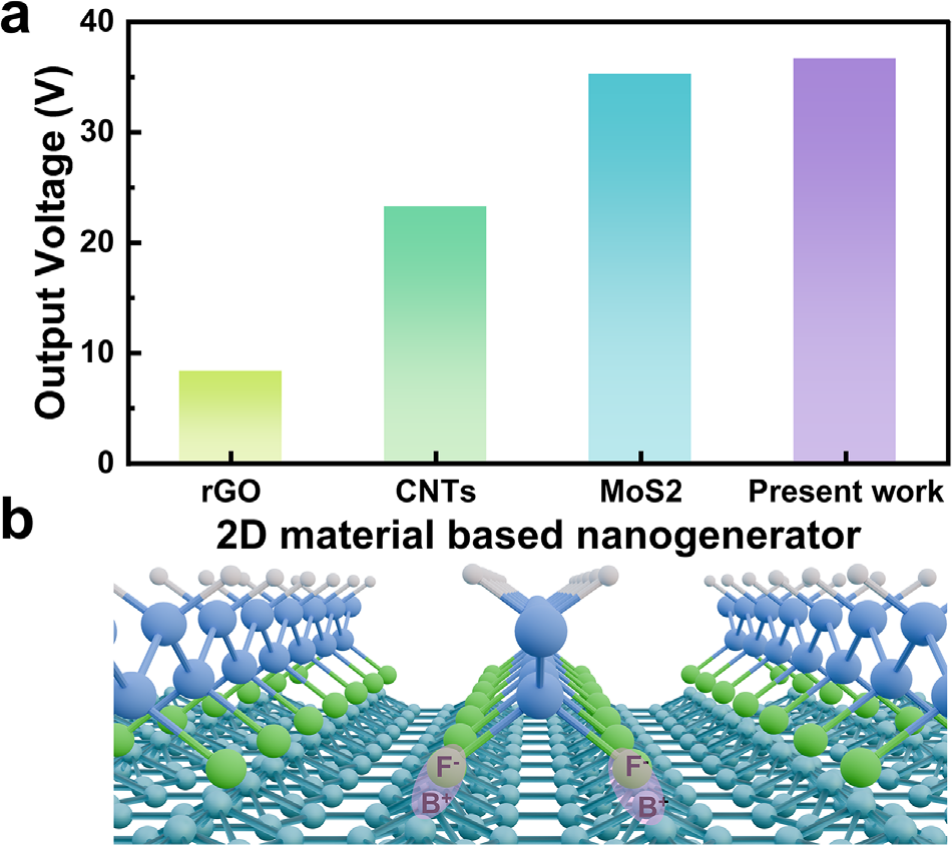
a) Comparison of the output voltage of nanogenerators based on different 2D materials. b) Schematic illustration of the interactions between PVDF and borophene, showing the alignment of PVDF dipoles facilitated by borophene, which enhances the β‐phase content and piezoelectric properties of the composite.

The present research marks the first report on borophene‐based piezoelectric and triboelectric devices, showcasing remarkable and promising performance for future applications in wearable electronics. A comparative analysis has been illustrated, highlighting the superior performance of borophene‐based devices developed in this study relative to those utilizing other 2D materials such as graphene,^[^ ^41^ ^]^ CNTs,^[^ ^42^ ^]^ and MoS₂^[^ ^43^ ^]^ (**Figure** **6**a). While graphene is semi‐metallic with least charge density of states, borophene is metallic in its prominent phases. On the other hand, MoS_2_ and CNTSs are semiconducting. In contrast to graphene, CNTs, and borophene, MoS_2_ has poor flexibility due to lower Young's modulus. Moreover, MoS_2_ is brittle. Apart from the intrinsic electronic character and their elastic behavior, interactions of these quantum materials with PVDF matrix are crucial in determining piezoelectric/triboelectric performances of the nanocomposites. The interaction between the PVDF polymer and borophene opens new possibilities for developing composite materials with unique properties, including enhanced mechanical strength, piezoelectric performance, electrical conductivity, and thermal conductivity. These advanced composites hold significant potential for applications in flexible electronics, energy storage, thermoplastics, sensors, and actuators. The nature and extent of borophene‐PVDF interactions, as depicted in Figure 6b, are influenced by factors such as dispersion, interfacial chemical bonding, and the specific structural configurations of the materials, making this an exciting area for future research and innovation. Further, adequate functionalization of borophene could enhance interfacial bonding, thereby improving the structural integrity and stability of the composite under mechanical and thermal stress. This highlights the potential of borophene‐PVDF composites as a versatile platform for next‐generation materials and device engineering.

## 3 Conclusion

In this study, borophene was synthesized for the first time via a cryo‐exfoliation method using liquid nitrogen to freeze and thermally shock boron crystals. This process, driven by cryo‐quenching‐induced thermal stresses, effectively loosened inter‐layer coupling in boron crystals, enabling their exfoliation into borophene sheets through subsequent mild sonication. The resulting borophene comprised monolayer and few‐layer sheets with lateral dimensions ranging from 0.1 to 5 µm, as confirmed by AFM imaging. The cryo‐exfoliation synthesis approach is scalable, rapid, reproducible, and environmentally friendly, requiring no harsh chemicals. Characterization by Raman spectroscopy and XPS revealed excellent phase purity with minimal surface oxidation, while HRTEM imaging highlighted Moiré patterns indicative of layer stacking with rotational and translational offsets. Borophene was integrated as a nanofiller into PVDF matrices to demonstrate practical applications for creating nanocomposites. These composites exhibited exceptional piezoelectric and triboelectric performance, achieving voltage outputs of up to 57.8 V under mechanical stimuli. The findings establish borophene as a promising material for self‐powered wearable electronics and energy‐harvesting devices. This work pioneers a scalable and green synthesis method for borophene, opening new avenues for its application in advanced energy systems, sensing, and flexible electronics. Since borophene is the lightest among p‐block Xenes, is metallic in nature, and is chemically and electrochemically active, it holds immense potential in energy generation (batteries), energy storage (supercapacitors), electrocatalytic hydrogen evolution reaction (HER), oxygen evolution reaction (OER), ammonia reduction, and water splitting. Moreover, borophene's excellent anchoring capability for biomolecules and cells makes it suitable for biosensing and biomedical applications.

## 4 Experimental Section

### Materials

Crystalline boron powder (catalog no. BM1288) with 99% purity and an average particle size of ≈15 µm was purchased from Stanford Advanced Materials. Isopropyl alcohol (IPA) and N‐methyl‐2‐pyrrolidone (NMP) solvents were obtained from Sigma–Aldrich.

### Cryo‐Mediated Exfoliation of Borophene

The cryo‐mediated exfoliation of borophene was carried out using a combination of cryogenic cooling and solvent‐assisted sonication. The exfoliation process began by placing 200 mg of crystalline boron powder (catalog no. BM1288, with 99% purity and a particle size of ≈15 µm) into a 1 L plastic bottle. Liquid nitrogen was poured into the bottle filling approximately two‐thirds of its volume. The boron powder was immersed and rapidly cooled to cryogenic temperatures (≈−196 °C). The mixture was subjected to bath sonication, during which the liquid nitrogen evaporates. After the liquid nitrogen entirely evaporated during sonication, the bottle was refilled with liquid nitrogen. This process was repeated three times to ensure effective exfoliation. Afterward, 20 mg of the obtained solid was dispersed into a mixed solution of 20 mL isopropanol (IPA) and 20 mL N‐methyl‐2‐pyrrolidone (NMP) and sonicated by bath ultrasonicator (Cole Parmer 08895‐83) at power of 600 W for 30 min. The resulting suspension was centrifuged at 4500 rpm for 5 min, and the supernatant containing the exfoliated borophene was collected. This method offers several advantages over traditional exfoliation techniques, such as ease of operation and the ability to produce high yields of few‐layer borophene. The cryogenic temperatures and subsequent sonication steps help preserve the atomic structure of borophene while ensuring efficient layer separation.

This step was crucial in weakening the interlayer interactions between boron atoms, as the thermal shock caused by rapid cooling induces stress within the material. To enhance this effect, the boron bottle and liquid nitrogen were placed into a bath sonicator. The sonication process caused the liquid nitrogen to boil and evaporate within minutes, further aiding in the disruption of boron layers. This cryogenic treatment was repeated three times to ensure adequate pre‐exfoliation of the boron powder, which helped to loosen the interlayer bonds and prepare the material for further exfoliation.

After the cryogenic treatment, the remaining solids were collected and subjected to a solvent‐assisted exfoliation process. A portion of the cryogenically treated boron powder (20 mg) was transferred to a 40 mL glass vial. To this, 20 mL of IPA and 20 mL of NMP were added, creating a suspension. Using these solvents was critical, as they have been shown to stabilize exfoliated 2D materials effectively. The mixture was then sonicated for 30 min at room temperature. During this sonication step, the mechanical energy from the ultrasonic waves facilitated the further exfoliation of borophene sheets by breaking down the weakened interlayer interactions within the boron powder.

Once sonication was complete, the mixture was centrifuged at 4500 rpm for 5 min to separate unexfoliated boron particles from the exfoliated borophene sheets. After centrifugation, the supernatant containing the exfoliated borophene in suspension was carefully collected. This suspension represented the final exfoliated borophene product, which was subsequently used for further characterization. The combination of cryogenic treatment and solvent‐assisted sonication proved to be an effective method for producing borophene sheets with high structural integrity and minimal defects.

### Characterization

The samples were dispersed in IPA and drop‐cast on the lacey carbon copper grids. After drying and vacuuming in a dry pump station, the TEM images were taken by a JEOL JEM‐F200 Multi‐purpose Electron Microscope at 200 kV under high vacuum (HV) conditions. Drop casted dispersion of borophene on thoroughly cleaned borosilicate glass substrates and adequately dried thereafter, were used to capture AFM images (AFM; Multimode scanning probe Microscope, Bruker) in non‐contact dynamic force mode (DFM). Raman spectroscopy was carried out employing a STR 500 confocal microraman spectrometer equipped with second harmonic Nd‐YAG laser (532 nm). XPS spectroscopy was accomplished under ultrahigh vacuum (UHV) conditions in an ESCA+ Omicron NanoTechnolgy instrument and was employed to precisely determine the chemical composition of synthesized borophene samples.

### Molecular Dynamics Simulation

To consider exfoliation of borophene sheets, a bonded bilayer borophene van der Waals layer was considered.^[^ ^44^ ^]^ Constant temperature (475 K) Born Oppenheimer Molecular Dynamics (BOMD) simulations were performed considering the canonical ensemble (NVT) as implemented in the Vienna Ab initio Simulation Package (VASP).^[^ ^45^, ^46^, ^47^ ^]^ A 320 atom borophene layered supercell was used for BOMD simulations with an increased c lattice parameter (50 Angstrom). During the simulations, a Nose‐Hoover thermostat was used to adjust the temperature at 475 K (≈201 degree Celsius), and a time step of 1 fs was considered to integrate the equation of motion.^[^ ^48^, ^49^ ^]^ The simulation was run up to 50 ps to observe the exfoliation process. These simulations suggest a increment of a bilayer distance from 4.1 Angstrom to 6.9 Angstrom in 50 ps.

### Synthesis of Cryo‐B/PVDF Composite Thin Films

The synthesis of Cryo‐B/PVDF composite films involved integrating cryo‐exfoliated borophene (Cryo‐B) as a nanofiller into a PVDF polymer matrix. Initially, 1 gram of PVDF was dissolved in 20 mL of N, N‐dimethylformamide through continuous stirring at room temperature until a homogenous solution was achieved. Subsequently, Cryo‐B powder was added to the solution at a concentration of 5% by weight relative to PVDF. The mixture was stirred at 70 °C for 24 h to ensure uniform dispersion of the Cryo‐B particles within the PVDF solution. To further enhance homogeneity, the mixture underwent 30 min of sonication. This step facilitated an even distribution of Cryo‐B nanoparticles by breaking down any agglomerates. Using a solution casting technique, the resulting mixture was poured into petri dishes and dried at 80 °C for 6 h in a vacuum oven to produce flexible, free‐standing composite thin films. The dried films were then cut into 1 × 1 cm^2^ pieces for further analysis. These Cryo‐B/PVDF films were subjected to structural, mechanical, and electrical characterizations and piezoelectric energy harvesting tests, demonstrating their potential for flexible and wearable energy devices. This method ensures a scalable and reproducible approach for fabricating high‐performance Cryo‐B/PVDF nanocomposites.

## Conflict of Interest

The authors declare no conflict of interest.

## Supporting information



Supporting Information

## Data Availability

The data that support the findings of this study are available in the supplementary material of this article.
