# Supplementary material for: Cryo‐Exfoliation Synthesis of Borophene and its Application in Wearable Electronics
Source: Adv Sci (Weinh). 2025 Apr 4;12(26):2502257. doi: 10.1002/advs.202502257 (PMC12245121; doi:10.1002/advs.202502257)
Supplement: Supplementary file 1 — Supporting Information [file ADVS-12-2502257-s001.docx]

Supporting Information

Cryo-exfoliation synthesis of borophene and its application in wearable electronics

Zhixuan Li, Gaurav Pandey, Arkamita Bandyopadhyay, Kamlendra Awasthi, John V. Kennedy, Prashant Kumar*, Ajayan Vinu*





**Figure S1.** 50 K magnification TEM image of cryo-exfoliation borophene.





**Figure S2.** 50 K magnification TEM image of cryo-exfoliation borophene.





**Figure S3.** 100 K magnification TEM image of cryo-exfoliation borophene.





**Figure S4.** 100 K magnification TEM image of cryo-exfoliation borophene.





**Figure S5.** 200 K magnification TEM image of cryo-exfoliation borophene.





**Figure S6.** 200 K magnification TEM image of cryo-exfoliation borophene.





**Figure S7.** 400 K magnification TEM image of cryo-exfoliation borophene.


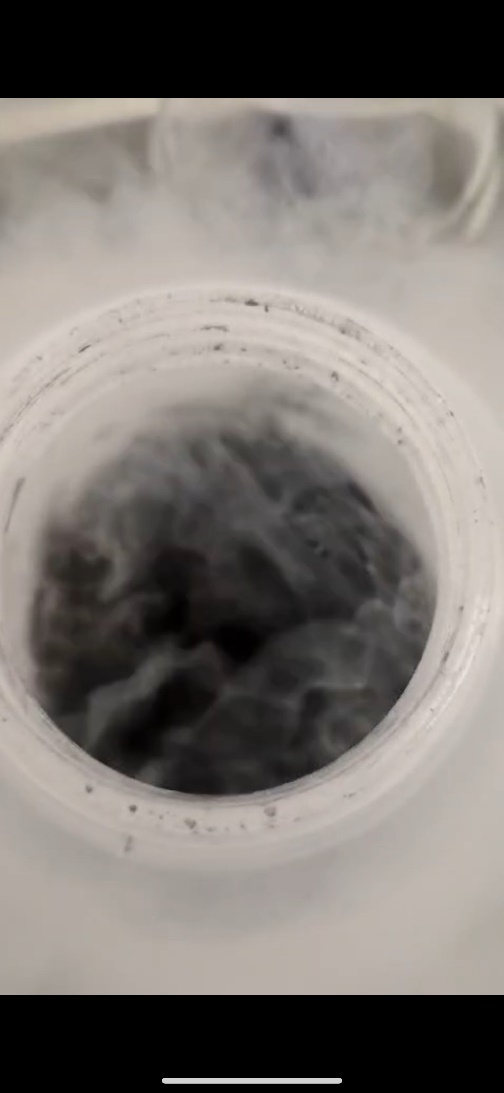

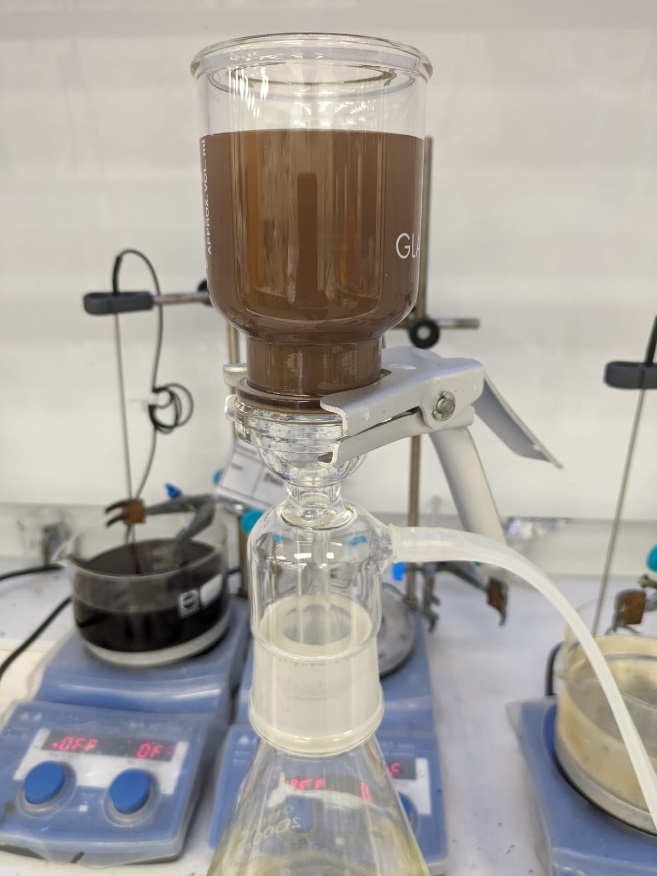


**Figure S8.** Photo of liquid nitrogen evaporation process and suspension of exfoliated borophene after centrifugation.
